# Therapeutic Drug Monitoring of Busulfan in Patients Undergoing Hematopoietic Cell Transplantation: A Pilot Single-Center Study in Taiwan

**DOI:** 10.3390/ph14070613

**Published:** 2021-06-26

**Authors:** Rong-Long Chen, Li-Hua Fang, Xin-Yi Yang, Mohsin El Amrani, Esther Veronique Uijtendaal, Yen-Fu Chen, Wei-Chi Ku

**Affiliations:** 1 Department of Pediatric Hematology and Oncology, Koo Foundation Sun Yat-Sen Cancer Center, Taipei City 112019, Taiwan; ronglongchen@yahoo.com.tw; 2 Department of Pharmacy, Koo Foundation Sun Yat-Sen Cancer Center, Taipei City 112019, Taiwan; fang500301@gmail.com (L.-H.F.); s21007@skmh.tmu.edu.tw (X.-Y.Y.); 3 Department of Clinical Pharmacy, Division Laboratory, Medicine and Pharmacy, University Medical Center Utrecht, Utrecht University, 3584 CX Utrecht, The Netherlands; M.ElAmrani@umcutrecht.nl (M.E.A.); E.V.Uijtendaal@umcutrecht.nl (E.V.U.); 4 School of Medicine, College of Medicine, Fu Jen Catholic University, New Taipei City 242062, Taiwan; cytftofu@hotmail.com

**Keywords:** busulfan, therapeutic drug monitoring, hematopoietic cell transplantation

## Abstract

Busulfan has been used as a conditioning regimen in allogeneic hematopoietic cell stem transplantation (HSCT). Owing to a large inter-individual variation in pharmacokinetics, therapeutic drug monitoring (TDM)-guided busulfan dosing is necessary to reduce graft failure and relapse rate. As there exists no TDM of busulfan administration for HCT in Taiwan, we conducted a pilot study to assess the TDM-dosing of busulfan in the Taiwanese population; Seven patients with HCT from The Koo Foundation Sun Yat-Sen Cancer Center, Taipei, Taiwan, received conditioning regimens consisting of intravenous busulfan and other chemotherapies. After the initial busulfan dose, blood samples were collected for busulfan TDM at 5 min, 1 h, 2 h, and 3 h. Busulfan was extracted and detected by performing stable-isotope dilution LC–MS/MS. Plasma busulfan concentration was quantified and used for dose adjustment. Potential adverse effects of busulfan, such as mucositis and hepatic veno-occlusive disease (VOD), were also evaluated; The LC–MS/MS method was validated with an analyte recovery of 88–99%, within-run and between-run precision of <15%, and linearity ranging from 10 to 10,000 ng/mL. Using TDM-guided busulfan dosing, dose adjustment was necessary and performed in six out of seven patients (86%) with successful engraftments in all patients (100%). Mild mucositis was observed, and VOD was diagnosed in only one patient; This single-center study in Taiwan demonstrated the importance of busulfan TDM in increasing the success rate of HCT transplantation. It is also necessary to further investigate the optimal busulfan target value in the Taiwanese population in the future.

## 1. Introduction

Allogeneic hematopoietic cell transplantation (HCT) is a therapeutic approach used for managing several hematopoietic or genetic diseases [1]. For example, successful allogeneic HCTs have been reported in the treatment of malignancies, such as acute myeloid leukemia (AML) [2], neuroblastoma [3,4], and inborn genetic errors such as adrenoleukodystrophy (ALD) [5] and Lesch–Nyhan syndrome (LNS) [6,7].

Before HCT is conducted in recipients, conditioning regimens are administered to reduce the occurrence of tumors and immune ablation. Traditionally, a high dose of total body irradiation has been used for HCT conditioning; however, patients usually present with immediate or delayed toxicities [1]. Alternatively, chemotherapy-based conditioning was introduced in the 1980s to replace total body irradiation [2]. Currently, busulfan, an alkylating agent, combined with other chemotherapeutic agents, such as fludarabine, is the most widely used conditioning regimen for allogeneic HCT [8,9,10].

Since the 1950s, oral busulfan has been used as an effective treatment for chronic myelogenous leukemia and other hematopoietic malignancies [11]. Owing to the differences in absorption, administration of the oral form of busulfan often results in substantial variability in plasma levels between patients, thereby limiting its use as an HCT conditioning agent [12]. With the introduction of intravenous busulfan, the pharmacokinetic variations between patients have reportedly reduced; therefore, the safety of the regimen has increased [13]. However, a considerable inter-individual variation of busulfan concentration in plasma, especially in children and young adults, has been reported [14].

In addition, drug–drug interactions (DDIs) may affect the clearance of busulfan or co-administered drugs [15,16,17]. Using the pharmacokinetic (PK) interaction network-based molecular structural similarity, Hao et al. predicted six clinically relevant and literature-reported DDIs for busulfan, including voriconazole, fludarabine, itraconazole, cyclophosphamide, metronidazole, and melphalan [15]. For example, a combination of busulfan with fludarabine, cyclophosphamide, or melphalan has been reported as a standard conditioning regimen for HCT [18,19]. The considerably decreased clearance of melphalan or cyclophosphamide results in potentially lethal toxicity when pre-administered with busulfan [20]. Therefore, administration of busulfan after cyclophosphamide or melphalan may prevent a decrease in the clearance of both drugs and reduce the risk of hepatotoxicity. Although the DDIs between busulfan and fludarabine have not been consistently established, fludarabine is also administered before busulfan. Itraconazole, voriconazole, or metronidazole can either be replaced by other drugs that do not have DDIs with busulfan or be administered after completion of busulfan treatment, thus preventing a reduction in busulfan clearance.

To further reduce the busulfan pharmacokinetic variations between patients and potential DDIs, therapeutic drug monitoring (TDM) was prescribed to guide the administration of intravenous busulfan doses [21,22]. Since steady-state busulfan concentrations in plasma can be predicted from first-dose kinetics, TDM-based busulfan dosing in HCT conditioning substantially improves event-free survival in children [23,24,25,26]. In Taiwan, busulfan combined with other chemotherapies [26] is commonly used for HCT conditioning. However, whether TDM-guided busulfan administration can increase the success rate of transplantation and event-free survival in patients remains unknown. In this study, we demonstrated that busulfan pharmacokinetics differed between patients, and nearly all patients were subjected to dose adjustment using TDM data obtained from the analysis of the first dose of busulfan. With TDM-guided busulfan dosing, the success rate of stem cell engraftment substantially increased. Our data suggest the need for busulfan TDM for HCT conditioning in Taiwan.

## 2. Results

### 2.1. Recovery and Matrix Effect

Several LC–MS/MS methods have been reported for the quantitation of busulfan in biological fluids [27,28,29,30,31,32]. Recently, Punt et al. developed an LC–MS/MS method for the simultaneous quantitation of busulfan, clofarabine, and fludarabine in plasma [28]. This method has been applied to study the population PKs of fludarabine and clofarabine [33,34,35]. The combination of busulfan and fludarabine, with or without clofarabine, is currently emerging as an HCT conditioning regimen [36]. In this study, we also performed HCT conditioning by combing busulfan with fludarabine; therefore, we decided to adopt Punt’s method [28] and focus on busulfan TDM.

**Figure 1:**
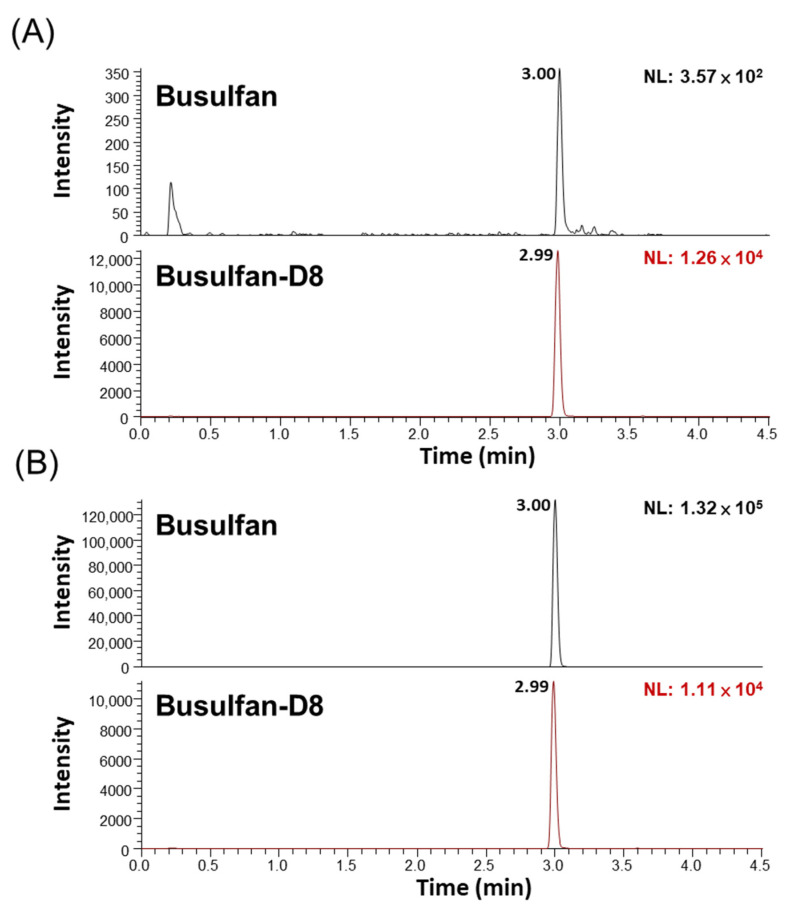
Representative elution chromatograms of busulfan and busulfan-D8 at different levels using newborn calf serum. (**A**) Lower limit of quantitation (LLOQ) at 10 ng/mL busulfan, and (**B**) medium QC (MQC) at 4000 ng/mL busulfan. The SRM transitions were busulfan (264 > 151 *m*/*z*, 264 > 151 *m*/*z*) and busulfan-D8 (272 > 159 *m*/*z*, 272 > 62 *m*/*z*).

First, the elution profile of both busulfan and busulfan-D8 internal standards showed good peak shapes (Figure 1). Subsequently, the recovery and matrix effects were assessed. Table 1 depicts the recoveries of QC low (LQC) and QC high (HQC) levels as 92.34% and 99.54%, respectively. Additionally, the matrix effect, as evaluated using the matrix factor procedure, ranged from 88.53% to 99.32% (Table 1). Together, these data suggest the efficient extraction of busulfan from plasma/serum with minimal matrix interference.

### 2.2. Linearity

**Figure 2:**
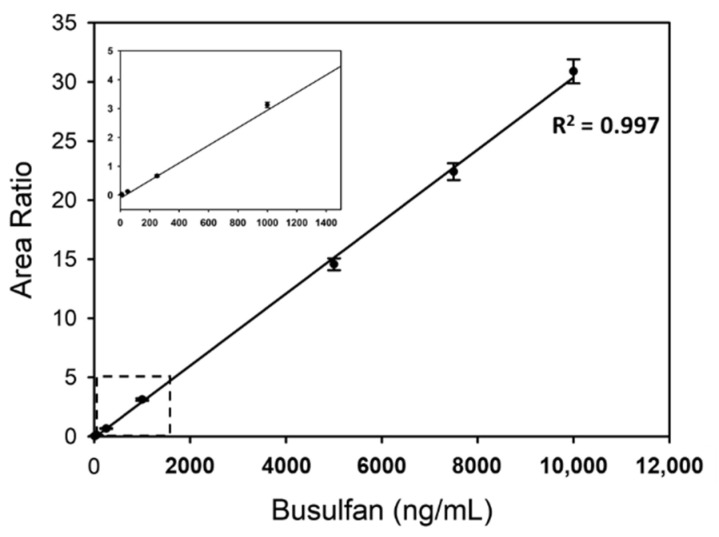
Linearity of busulfan analysis using newborn calf serum with 10, 50, 250, 1000, 5000, 7500, and 10,000 ng/mL busulfan. For each concentration, mean values from 10 repeated results were plotted. The region of the dashed-line box was enlarged in a small plot. The coefficients of determination R^2^ of the trend line have been also presented.

A calibration curve of busulfan ranging from 10 to 10,000 ng/mL has been shown in Figure 2. The equation of the calibration curve was *y* = 0.0030*x* − 0.1059, where *y* represents the area ratio and *x* the busulfan concentration. The coefficient of determination R^2^ was 0.997 in the linear range, which is considered enough for the analysis of clinical sample concentrations empirically ranging from 200 to 5000 ng/mL [29,37].

### 2.3. Precision and Accuracy

The precision and accuracy of LQC, MQC, and HQC are listed in Table 2. The within-run and between-run precision and accuracy rates were <15%, which were within acceptable levels. Since the busulfan concentration in patients obtaining conditioning regimens was considerably higher than the LOQ, we used a calibration curve of busulfan concentrations ranging from 50–7500 ng/mL in clinical samples.

### 2.4. Proficiency Test

Plasma samples from three patients were analyzed in parallel by two laboratories (UMC Utrecht in The Netherlands [28] and FJCU in Taiwan) to estimate inter-laboratory differences. As shown in Table 3, differences in busulfan concentrations at the four time points in three patients were between 11.53 and 6.73%, and the average differences (mean ± standard deviation) were 0.71 ± 6.17% in patient A, 3.49 ± 3.88% in patient B, and 7.77 ± 3.14% in patient C. The difference in the calculated cumulative area under the concentration–time curve (cAUC) for the patients was also less than 7% between the two laboratories. The results demonstrate the robustness of the method.

### 2.5. Clinical Samples

In this study, a total number of seven patients requiring HCT were included. Their disease, age, sex, and clinical information have been summarized in Table 4. The initial busulfan dose in co-conditioning regimens was administered according to the EBMT/ESID guidelines for ablative HSCT [26], and the following conditioning on day 4 was adjusted based on the busulfan PK from the calculated cAUC data on day 1 (Table 4). The target value of busulfan exposure was 90 mg × h/L, which was based on a multicenter retrospective cohort analysis [25]. Busulfan dose adjustments were advised for all patients except patient 6 because a cAUC difference of <5% from the optimal cAUC (90 mg × h/L) does not require a dose adjustment (Table 4) [25]. With personalized busulfan-based conditioning, all patients (100%) showed successful neutrophil and platelet engraftments, which were defined as an absolute neutrophil count over 0.5 × 10^9^/L in the first 3 consecutive days and platelet counts over 2 × 10^10^/L without platelet transfusion in the first 7 consecutive days, respectively.

Additionally, we determined potential and acute adverse effects after three weeks of successful engraftment, such as mucositis [38] and hepatic veno-occlusive disease (VOD) [39]. Most patients had mild mucositis (grade 1 or 2), and VOD was only diagnosed in patient 7 (Table 4). We also monitored the endpoint of survival, which was determined by a minimum follow-up of six months [25]. All patients, except patient 2, were alive without presentation of major organ complications after six months. Collectively, the data demonstrated that TDM-guided busulfan-based conditioning is important for successful engraftment in Taiwan.

## 3. Discussion

When used in HCT conditioning, the busulfan cAUC has an extremely narrow therapeutic window. For example, a target busulfan cAUC (100 mg × h/L) in combination with fludarabine is necessary for the US adult population and a higher cAUC results in worse clinical outcomes [40]. On the other hand, a low busulfan cAUC is associated with graft rejection and disease relapse, especially in pediatric HCT transplantation [24,41]. Based on a recent comprehensive, retrospective study in children and young adults, the busulfan cAUC of 78–101 mg × h/L showed the best clinical outcome [25]. These data are in agreement with a target cAUC of 90 mg × h/L for myeloablative exposure, as suggested by the EBMT/ESID [42]. Therefore, it is important to perform busulfan TDM to achieve an optimal HCT outcome.

Based on the analysis of clinical samples, to the best of our knowledge, this is the first busulfan TDM study to report the application of personalized busulfan conditioning for HCT in Taiwan. Currently, there are no PK studies of busulfan in Taiwan. Although the busulfan target cAUC of 90 mg × h/L used in this study was originally intended for children and young adults in Western populations [25,42], we found that HCT engraftments were successful in all patients receiving this target value (Table 4). For adult patients (nos. 3 and 4), patient no. 4 had the best event-free prognosis up to February 2021. The other patient (no. 3) succumbed to pneumonia, which was not directly associated with busulfan exposure. As for the children (patient nos. 2, 5, 6, and 7) and young adults (patient no. 1), patient no. 4 had the best event-free survival up to February 2021.

It was noted that patient 2, with LNS, developed infection-associated multi-organ failures, which may have resulted from an interaction between germline defects and DDIs. It was further noted that patient 7 had many risk factors associated with VOD, including underlying relapsed/refractory neuroblastoma, serologically positive anti-hepatitis B core antibody, high dose melphalan use, as well as prior partial hepatectomy. Therefore, potential DDIs were suspected. He developed liver VOD according to the EBMT diagnostic criteria [43,44], although this was resolved by supportive management. 

This study had some limitations. Owing to the limited number of patients investigated in this study, we could not determine whether the busulfan target cAUC of 90 mg × h/L was optimal for event-free survival in the Taiwanese population. TDM-guided busulfan conditioning is only part of the regimen for successful treatment. Knowledge of the underlying diseases in patients and highly specialized care are required to achieve the best event-free outcomes for patients with HCT [45]. Nevertheless, the clinical results presented in this study highlight the importance of busulfan TDM in HCT transplantation, especially in Taiwan. Further studies are warranted to obtain a potentially optimal busulfan target cAUC for the Taiwanese population.

## 4. Materials and Methods

### 4.1. Chemicals and Reagents

Busulfan-D8 stock solution (100 μg/mL in methanol) was purchased from Cerilliant (Round Rock, TX, USA). Newborn calf serum was obtained from Thermo Fisher Scientific (Waltham, MA, USA). All other chemicals were purchased from Sigma-Aldrich (St. Louis, MO, USA).

### 4.2. Patients and Samples

Patients included in this study were admitted at The Koo Foundation Sun Yat-Sen Cancer Center (KFSYSCC), Taipei, Taiwan, from December 2015 to June 2017. The patients received an HCT conditioning regimen consisting of intravenous, pharmacokinetically dosed busulfan combined with fludarabine, melphalan, or fludarabine/clofarabine. The initial busulfan dose in co-conditioning regimens was administered according to the European Society for Blood and Marrow Transplantation/European Society for Immunodeficiencies guidelines for ablative HSCT [26]. Blood samples from each patient were collected for busulfan TDM at the indicated times (5 min, 1 h, 2 h, and 3 h) after busulfan infusion [28]. After centrifugation, plasma samples were obtained, aliquoted, and stored at −80 °C until analysis. Potential adverse effects of busulfan, such as mucositis and hepatic VOD, were evaluated as per previously described methods [43,44]. 

### 4.3. Preparation of Standards Internal Standard and Quality Control (QC) Samples

Calibration standards and QC samples were prepared in newborn calf serum according to a previously described method [28]. Although it is preferred to use blank human plasma, the busulfan-D8 internal standard (ISTD) in new calf serum and normal human plasma showed comparable internal standard matrix factors [28], which is acceptable based on the European Medicines Agency (EMA) guidelines. In brief, a busulfan stock solution (1 mg/mL) was freshly prepared in a 2 mL volumetric flask with N, N-dimethylacetamide, which is a solvent used in the preparation of intravenous busulfan [13,28]. Calibration standards at concentrations of 10, 50, 250, 1000, 5000, 7500, and 10,000 ng/mL busulfan were prepared by diluting the busulfan stock solution with the newborn calf serum in 2 mL volumetric flasks [28]. Different levels of quality control (QC) at low (25 ng/mL) (LQC), medium (4000 ng/mL) (MQC), and high (8000 ng/mL) (HQC) concentrations were also prepared in the newborn calf serum with 2 mL volumetric flasks [28]. Separate busulfan stock solutions were prepared to calibrate standards and QC samples. The calibration standards and QC samples were aliquoted and stored at −80 °C until use. The busulfan-D8 ISTD working solution was prepared by diluting the stock solution to 1 μg/mL in 50% acetonitrile (ACN) in a 2 mL volumetric flask and stored at 4 °C. 

### 4.4. Sample Preparation

In a 1.5 mL microcentrifuge tube, 50 μL plasma samples (standards, QC, and patient samples) were mixed with the following reagents in sequence: 12.5 μL ISTD working solution, 12.5 μL of 50% ACN, and 25 μL of 20% trichloroacetic acid (20%), as per previously described methods [28]. The mixtures were vortexed for 60 s and centrifuged for 5 min at 10,000× *g*. The supernatant (60 μL) was transferred into a new glass vial containing 540 μL of 5% ACN. The samples were analyzed by performing LC–MS/MS 24 h after preparation.

### 4.5. LC–MS/MS

The prepared samples were analyzed using the TSQ Quantiva triple quadrupole mass spectrometer (Thermo Fisher Scientific), which was coupled on-line with the UltiMate 3000 Open Sampler XRS System (Thermo Fisher Scientific). LC separation was performed as per methods previously described [28]. Eight microliters of the sample were loaded into an ACQUITY UPLC BEH C18 Column (130 Å, 1.7 µm, 2.1 × 50 mm; Waters, Milford, MA, USA). Mobile phase A comprised 0.1% formic acid in water, and mobile phase B included acetonitrile with 0.1% ammonium acetate. The elution program was conducted 0–2.4 min in isocratic 5% B, 2.4–2.75 min from 5% to 95% B, 2.75–3.5 min in isocratic 95% B, 3.5–4.0 min from 95% to 5% B, and 4.0–4.5 min in isocratic 5% B, with a flow rate 0.7 mL/min throughout the analyses. The ion source parameters were as follows: sheath gas of 38, aux gas of 12, ion transfer tube temperature of 250 °C, and vaporizer temperature of 279 °C. Busulfan and Busulfan-D8 were analyzed in the selected reaction monitoring mode with the following transitions: Busulfan 264 > 151 *m*/*z* (quantifier) and 264 > 151 *m*/*z* (qualifier) (CE: 10 V, RF: 50 V), as well as Busulfan-D8 272 > 159 *m*/*z* (quantifier) (CE: 11 V, RF: 48 V) and 272 > 62 *m*/*z* (qualifier) (CE: 23 V, RF: 48 V). A dwell time of 50 ms was observed for both Q1 and Q3 resolutions (FWHM) of 0.7 Da, CID gas of 1.5 mTorr, source fragmentation of 10 V, and chrom filter of 3 s. 

### 4.6. Method Validation

Method validation was performed according to the EMA guidelines. Recovery was tested by comparing the amount of busulfan in the plasma before and after extraction (before/after). The matrix effect was evaluated using the matrix factor procedure by adding the same amount of busulfan to plasma or water (neat) followed by sample extraction. Both recovery and matrix effects were tested in duplicate at the LQC and HQC. The freeze-and-thaw (five cycles) sample stability was assessed in triplicate at LQC and HQC. For accuracy and precision testing, six replicate QC samples at LQC, MQC, and HQC were assayed in one batch, and three batches on consecutive days were assayed. For the proficiency test, aliquots of plasma samples obtained from the same patient (total patient number = 3) were both assayed in Taiwan and The Netherlands within 3 days. 

### 4.7. Busulfan Exposure Calculation and Dose Adjustment

Busulfan exposure in individual patients was calculated as cAUC using a Bayesian fitting procedure with the PK Software (MW Pharm, Mediware, Zuidhoorn, The Netherlands) and a validated population PK model, as per previously described methods [25]. A dosing nomogram was used to determine the initial dose for each patient. Blood samples were collected for busulfan TDM at the indicated time points (t = 5 min, 1 h, 2 h, and 3 h after busulfan infusion) on day 1, and the cAUC was calculated using the validated population PK model. Further dose adjustment was based on the cAUC determined by the PK data to attain a target cAUC of 90 mg × h/L [25].

## 5. Conclusions

In conclusion, we applied LC–MS/MS-based busulfan TDM to personalized busulfan conditioning for HCT in seven patients. This pilot, single-center study demonstrated the importance of busulfan TDM for the optimal success rate of HCT transplantation. Expansion of the cohorts for busulfan TDM, establishment of the pharmacokinetic model, and the optimal busulfan exposure in the Taiwanese population are vital.

## Figures and Tables

**Table 1:** Recovery and matrix effect tests in QC low (LQC) and high (HQC) levels.

Busulfan Level	Concentration (ng/mL)	Recovery (%) *n* = 2	Matrix Factor (%) *n* = 2
LQC	25	92.34	88.53
HQC	8000	99.54	99.32

**Table 2:** Validation results for accuracy and precision.

Busulfan Level	Concentration (ng/mL)	Within-Run CV (%)	Between-Run CV (%)	Overall CV (%)	Overall Bias (%)
LQC	25	9.70	1.08	9.76	1.04
MQC	4000	2.54	1.19	2.78	2.18
HQC	8000	2.87	0.88	2.97	7.58

**Table 3:** Proficiency test between two laboratories.

Patient	Laboratory	Measured Busulfan Concentration (ng/mL)				cAUC ^4^ (mg × h/L)
		5 min	1 h	2 h	3 h	
A	UMC ^1^	5216	3886	3186	2479	29.2
	FJCU ^2^	4836	3995	3226	2658	29.3
	%Difference ^3^	−7.86	2.73	1.24	6.73	0.34
B	UMC	3946	2982	2318	1804	22.4
	FJCU	3871	3182	2398	1925	23.9
	%Difference	−1.94	6.29	3.34	6.29	6.28
C	UMC	5127	3247	2458	1935	23.8
	FJCU	4729	3124	2293	1735	22.4
	%Difference	−8.42	−3.94	−7.20	−11.53	−6.25

^1^ UMC: University Medical Center, Utrecht. ^2^ FJCU: Fu Jen Catholic University. ^3^ %Difference was determined by (FJCU-UMC)/UMC × 100%. ^4^ cAUC, cumulative area under the concentration–time curve.

**Table 4:** Clinical patient data.

No.	Underlying Diseases ^1^	Age (yrs)/Gender	Conditioning Regimen ^1^	BW ^1^-Guided Dose (mg) [26]	TDM ^1^-Guided Dose (mg) [25]	Difference ^2^ (%)	Mucositis	VOD ^1^
1	AML, relapsed	21/F	Flu + Bu	804	617	−5.2	Grade 2	None
2	LNS	1.5/M	Flu + Bu	178	142	−20.3	Grade 2	None
3	AML, relapsed	55/M	Flu + Bu + Clo	876	1047	19.5	Grade 1	None
4	AML	50/F	Flu + Bu	696	762	9.5	Grade 1	None
5	ALD	5/M	Flu + Bu	400	300	−25.0	Grade 2	None
6	AML	16/M	Flu + Bu	240	240	0.0	Grade 3	None
7	NB	7/M	Bu + Mel	360	420	16.7	Grade 2	Yes

^1^ Abbreviations: AML, acute myeloid leukemia; LNS, Lesch–Nyhan syndrome; ALD, adrenoleukodystrophy; NB, neuroblastoma; Flu, fludarabine; Bu, busulfan; Clo, clofarabine; Mel, melphalan; BW, body weight; TDM, therapeutic drug monitoring; VOD, veno-occlusive disease. ^2^ %Difference was determined by (TDM-BW)/BW × 100%.

## Data Availability

Data is contained within the article.
